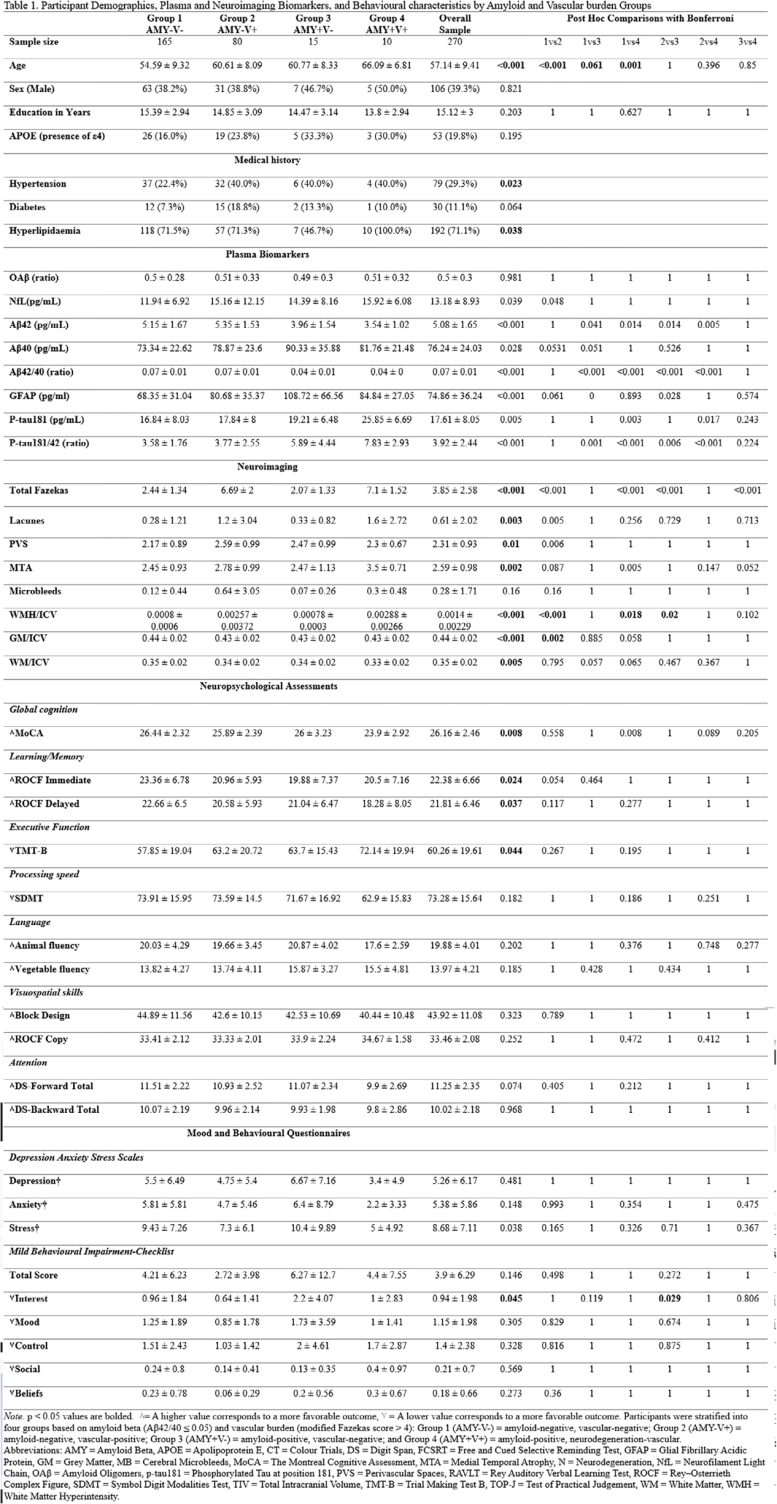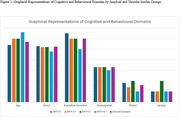# Deciphering the Prodrome: Synergistic Amyloid and Vascular Pathologies in Subjective Cognitive Decline – Insights from the Southeast Asian BIOCIS Study

**DOI:** 10.1002/alz70857_106197

**Published:** 2025-12-26

**Authors:** Yi Jin Leow, Justin Jit Hong Ong, Nagaendran Kandiah

**Affiliations:** ^1^ nil, nil, nil, Nicaragua; ^2^ Lee Kong Chian School of Medicine, Nanyang Technological University, Singapore, Singapore

## Abstract

**Background:**

Subjective Cognitive Decline (SCD) is increasingly recognized as a pivotal prodromal phase of Alzheimer's disease (AD), yet its underlying mechanisms remain elusive. Characterized by self‐reported cognitive impairment despite normal neuropsychological performance, SCD represents a crucial window for early intervention. This study investigates the interplay between amyloid pathology, cerebrovascular burden, and cognitive‐behavioral phenotypes in a Southeast Asian SCD cohort, leveraging fluid biomarkers, neuroimaging metrics, and neuropsychological assessments to delineate preclinical disease trajectories.

**Method:**

A total of 270 cognitively normal individuals with subjective memory complaints were stratified based on plasma amyloid‐beta (Aβ42/40≤0.05 indicating amyloid positivity) and vascular burden (modified Fazekas score>4 indicating high burden) into four groups: amyloid‐negative/vascular‐negative (A‐V‐), amyloid‐negative/vascular‐positive (A‐V+), amyloid‐positive/vascular‐negative (A+V‐), and amyloid‐positive/vascular‐positive (A+V+). Group differences were analyzed using ANOVA with Bonferroni corrections and chi‐square tests.

**Result:**

Distinct patterns emerged across amyloid and vascular burden groups. The A+V+group was the oldest (66.09±6.81 years), while the A‐V‐ group was the youngest (54.59±9.32 years, *p* <0.001). Cardiovascular risk factors, including hypertension and hyperlipidemia, were more prevalent in vascular‐positive groups (*p* = 0.023 and *p* = 0.038, respectively). GFAP levels were highest in A+V‐ and significantly elevated in A+V+(*p* <0.001 and *p* = 0.028), with *p*‐tau181/Aβ42 ratios peaking in A+V+(*p* <0.001).

Neuroimaging showed higher Fazekas scores in vascular‐positive groups (A‐V+ and A+V+, *p* <0.001), with lacunes and medial temporal atrophy most pronounced in A+V+(*p* = 0.003 and *p* = 0.002, respectively). Cognitively, A+V+ had the lowest global cognitive (MoCA) scores (*p* = 0.008), visuospatial memory performance (ROCF Immediate, Delayed Recall, *p* = 0.024 and *p* = 0.037) as well as executive functioning and processing speed (TMT‐B) (*p* = 0.044). Behavioral assessments revealed elevated stress levels (DASS) in A+V‐(*p* = 0.038) and greater apathy (MBI‐C‐interest‐subdomain) in A+V‐ compared to A‐V+(*p* = 0.029).

**Conclusion:**

This study highlights the impact of coexisting amyloid and vascular pathology in SCD, demonstrating that cognitive, structural, and behavioral differences between pathologies. A+V+ individuals exhibited the most severe cognitive impairments, underscoring the synergistic effects of amyloid and vascular burden. Meanwhile, behavioral findings suggest that amyloid positivity alone, as observed in A+V‐, may drive early emotional changes, potentially reflecting neural network disruptions associated with amyloid deposition.

The findings reinforce SCD as a key preclinical stage where integrating cognitive, biomarker, and behavioral assessments can help identify high‐risk individuals. Targeted interventions addressing vascular risk and neuropsychiatric symptoms may offer opportunities to slow progression to dementia.